# Evaluation of presentation, treatment and outcome in hypertensive emergency in dogs and cats: 15 cases (2003‐2019)

**DOI:** 10.1111/jsap.13530

**Published:** 2022-07-10

**Authors:** D. Beeston, R. Jepson, S. Cortellini

**Affiliations:** ^1^ Queen Mother Hospital for Animals Royal Veterinary College Brookmans Park AL9 7TA UK

## Abstract

**Objectives:**

Hypertensive emergency is well recognised in human medicine, yet there is limited veterinary evidence. This study aimed to determine the presentation, treatment and outcome in dogs and cats with hypertensive emergency.

**Materials and Methods:**

A retrospective case series of dogs and cats with hypertensive emergency identified as follows: acute history with non‐invasive Doppler systolic blood pressure greater than 180 mmHg and target organ damage including acute onset seizures, altered mentation with or without lateral recumbency or blindness. Data collected included signalment, history, physical examination and clinicopathological findings, systolic blood pressure, antihypertensive treatment and outcome.

**Results:**

Seven dogs and eight cats were included presenting with seizures (n=9), blindness (n=4), altered mentation with (n=2) or without (n=2) lateral recumbency. Median age was 9 years (range 1 to 15) and duration of clinical signs before presentation was 1.5 days (range 1 to 15). Median systolic blood pressure on presentation was 230 mmHg (range 190 to 300). Amlodipine was the most common first‐line agent (n=10), followed by hydralazine (n=4) and hypertonic saline (n=1). Aetiology of hypertensive emergency was acute kidney injury (n=9), idiopathic hypertension (n=3), hyperthyroidism (n=1), lymphoma (n=1) and suspected cutaneous and renal glomerular vasculopathy (n=1). Five cats and three dogs survived to discharge with an overall survival of 53.3%.

**Clinical Significance:**

Hypertensive emergencies had various presenting signs in this series. AKI was considered to be the cause of hypertension in the majority of patients. Further evaluation of treatment for hypertensive emergencies is warranted, considering almost half of the cases did not survive to discharge.

## INTRODUCTION

Arterial blood pressure is tightly regulated in all mammals through various neurohumoral, myogenic, metabolic and flow‐mediated processes (Magder 2018). Systemic hypertension has been associated with many conditions in veterinary species including acute kidney injury (AKI) (Cole *et al*. 2020), chronic kidney disease (O'Neill *et al*. 2013a,b), diabetes mellitus (Herring *et al*. 2014), immune‐mediated haemolytic anaemia (Hall *et al*. 2022) and pheochromocytoma (Gilson *et al*. 1994) in dogs, and most commonly chronic kidney disease (Conroy *et al*. 2019), hyperthyroidism (Williams *et al*. 2013) and primary hyperaldosteronism (Ash *et al*. 2005) in cats.

Hypertensive crisis is defined in human medicine as systolic blood pressure (SBP) or diastolic blood pressure (DBP) greater than 180 or 120 mmHg, respectively. Hypertensive crisis is then further classified into hypertensive emergency or hypertensive urgency based on the presence, or absence, of target organ damage (TOD) (Rodriguez *et al*. 2010). The risk of TOD in the presence of systemic hypertension has been well defined in veterinary medicine and includes damage to the kidneys, eyes, brain and heart (Acierno *et al*. 2018). Overt TOD in dogs and cats may most readily be recognised in the presence of ocular (blindness, retinal detachment or haemorrhage, hyphaema) or neurological (seizures, altered mentation) disease (Acierno *et al*. 2018).

Hypertensive emergency is rare in human medicine, accounting for only 0.2% of emergency department visits (Janke *et al*. 2016) and has an in‐hospital mortality of 12.5%, mostly attributed to neurovascular events with a median survival time of 14 days (Guiga *et al*. 2017). Whilst hypertensive urgency can be treated on an outpatient basis, hypertensive emergency requires immediate admission to an intensive care unit for prompt blood pressure control with titratable parenteral medications (Rodriguez *et al*. 2010). Treatment goals of hypertensive emergency include a reduction in mean arterial pressure by 20 to 25% in the first hour, followed by a gradual reduction to an SBP/DBP of 160/100 to 160/110 mmHg over 2 to 6 hours and further decrease to normotension over the following days (Rodriguez *et al*. 2010; Whelton *et al*. 2018; Van Den Born *et al*. 2019). However, it is currently unknown whether this same treatment approach is indicated or appropriate in veterinary species.

Literature on hypertensive emergency in veterinary medicine is scarce (Brown *et al*. 2005; O'Neill *et al*. 2013a,b; Church *et al*. 2019). The primary aim of this study was to describe the presentation, treatment and outcome of hypertensive emergency in a series of dogs and cats. The secondary aim was to evaluate the adherence to proposed human guidelines for the treatment of hypertensive emergency.

## MATERIALS AND METHODS

### Study design and inclusion criteria

This was a retrospective case series. Ethical approval was obtained by the Social Sciences Research Ethical Review Board at the author's institution (Queen Mother Hospital for Animals [QMHA], Royal Veterinary College). The electronic medical records of the QMHA were searched using a computerised search of dogs and cats presenting to the emergency and critical care service with hypertensive emergency.

Cases were eligible for inclusion if they had hypertensive emergency defined as an acute history with severe hypertension (SBP > 180 mmHg) and clinical signs consistent with neurological TOD including acute onset seizures, altered mentation present with or without lateral recumbency and blindness. All SBP measurements were performed using the non‐invasive Doppler technique or direct arterial measurement based on the equipment available at the institution. All cases presenting with blindness had fundoscopy performed to distinguish whether blindness was neurological or ophthalmic in origin. Cases were excluded if they were found to be non‐hypertensive or non‐emergent, or if they did not have serial blood pressure monitoring documented to confirm the diagnosis, or monitor progression, of hypertensive emergency.

### Medical record search

Candidate cases of hypertensive emergency were identified using search terms in the clinical notes (hypertensive encephalopathy, hypertensive seizures, hypertensive emergency, hypertensive crisis) and treatment fields (hydralazine, nitroprusside, fenoldopam, enalaprilat, phentolamine) relevant to the diagnosis and management of hypertensive emergency between January 1, 2003 and December 31, 2019. Search findings were merged and the clinical notes of all candidate cases were examined manually in detail to confirm whether they met the case definition.

### Data extraction

Data extracted from records included signalment, history, physical examination findings, clinical‐pathological findings, SBP, diagnostics performed, number and type of antihypertensive medications prescribed and survival to discharge, including modes of death. Fundoscopic examination was conducted by residents in training, under specialist supervision or specialists, from the Emergency and Critical Care, Internal Medicine and Ophthalmology services. Neurological examination was conducted by residents in training, under specialist supervision, or specialists from the Neurology service. SBP measurements were performed using the Doppler technique in accordance with ACVIM guidelines (Brown *et al*. 2007). Hospital records were assessed where available for compliance with human guidelines for hypertensive crisis management (Rodriguez *et al*. 2010; Van Den Born *et al*. 2019).

#### Statistical analysis

A commercial statistical software program was used for all analyses (SPSS Statistics, version 26; IBM). All continuous variables were evaluated for normality using the Shapiro–Wilk test. Normally distributed variables were described using mean (±sd) while median (range) was used to describe non‐normally distributed continuous variables. Categorical variables were described as frequencies and percentages.

## RESULTS

### Patient inclusion

**FIG 1:**
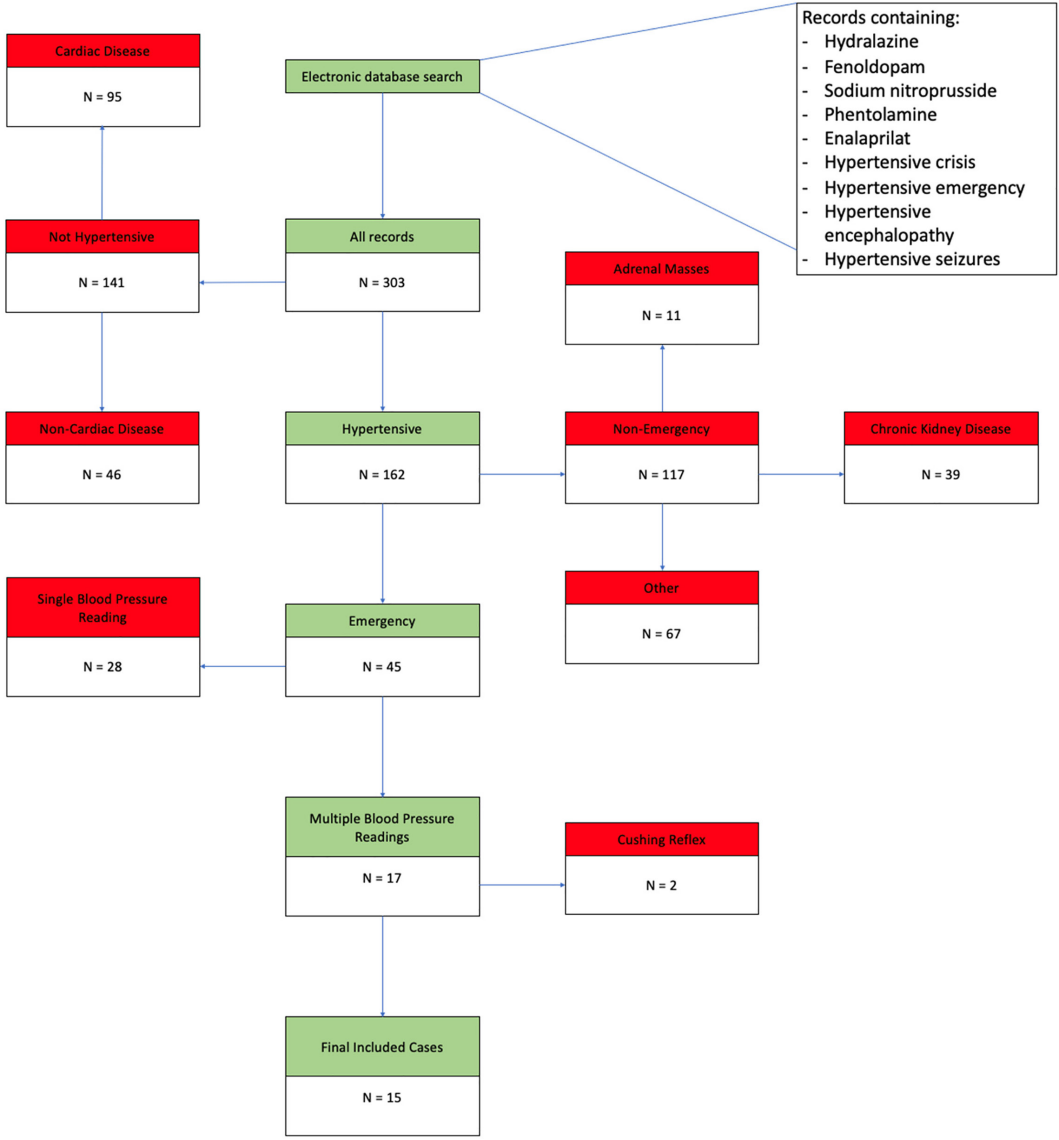
Results of medical record search between January 1, 2003 and December 31, 2019 for hypertensive emergency and subsequent patient inclusion

A total of 303 cases were identified using the search criteria (see Fig 1). Two hundred and fifty‐eight cases were excluded because they were found to be non‐hypertensive or non‐emergent, and 28 cases were excluded because only a single SBP evaluation on admission was written in the clinical records without further clinical records regarding SBP during hospitalisation. Seventeen cases met the inclusion criteria; nine dogs and eight cats. Two dogs were subsequently excluded following a review of the clinical records that suggested a Cushing reflex as the primary cause of hypertension. One dog was noted to have an intracranial mass, and the other dog was diagnosed with *Angiostrongylus vasorum* and intracranial haemorrhage. Fifteen cases remained for further analysis.

### Signalment

The study population consisted of seven dogs and eight cats. The dog population consisted of five of seven (71.4%) males [three of five (60%) neutered] and two of seven (28.6%) females [two of two (100%) neutered]. The cat population consisted of two of eight (25%) males [two of two (100%) neutered] and six of eight (75%) females [six of six (100%) neutered]. Dog breeds included one Yorkshire terrier, one Staffordshire bull terrier, one Airedale terrier, one Greyhound, one cocker spaniel and two crossbreeds. Cat breeds included five domestic short hairs, two domestic long hairs and one Burmese. Median age of dogs and cats were 7 years (range 1 to 11) and 12 years (range 5 to 15), respectively. In the dog population, median age of survivors and non‐survivors was 8.0 years (range 7 to 9) and 6.5 years (range 1 to 11), respectively. In the cat population, median age of survivors and non‐survivors was 14.0 years (range 9 to 15) and 8 years (range 5 to 12), respectively.

### Historical findings

Presenting complaints are summarised in Table 1. The median duration of clinical signs before presentation in all patients was 1 day (range 1 to 15). The median duration of clinical signs in survivors and non‐survivors was 1.5 days (range 1 to 10) and 1 day (range 1 to 15), respectively.

**Table 1:** Presenting complaint of referral patients diagnosed with hypertensive emergency between January 1, 2003 and December 31, 2019

Presenting complaint	Number of cases (%)
Dogs	7/15 (46.7%)
Blindness	3/7 (42.9%)
Altered mentation with lateral recumbency	2/7 (28.6%)
Seizures	2/7 (28.6%)
Cats	8/15 (54.3%)
Seizures	7/8 (87.5%)
Altered mentation without lateral recumbency	2/8 (25.0%)
Blindness	1/8 (12.5%)

### Diagnostics

A complete neurological examination was performed in 12 of 15 (80%) cases with abnormalities documented in 10 of 12 (83.3%) cases. Neurological abnormalities included absent menace response [bilateral six of 12 (50%), unilateral one of 12 (8.3%)], obtundation [two of 12 (16.6%)], anisocoria [two of 12 (16.6%)] and circling [two of 12 (16.6%)]. A complete fundoscopic examination was documented in 12 of 15 (80%) cases with abnormalities identified in nine of 12 (75%). Fundoscopic abnormalities included retinal detachment [eight of nine (88.8%)] and retinal haemorrhage [two of nine (22.2%)]. Fundoscopic findings in patients presenting with acute onset blindness [three of seven (42.9%) dogs and one of eight (12.5%) cats] included bilateral retinal detachment in three of four (75%) cases and retinal haemorrhage in one of four (25%) cases. In patients with an absent menace response, five of seven (71.4%) had retinal detachment.

Complete haematology and biochemistry performed on admission were available for review in 12 of 15 (80%) and 13 of 15 (86.7%) cases, respectively. Median creatinine in dogs and cats was 534 μmol/L (range 45 to 909) and 208.5 μmol/L (range 80 to 304), respectively. Median creatinine in dogs who survived was 96.5 μmol/L (range 45 to 148) and was 544 μmol/L (range 534 to 909) in dogs who did not survive. Median creatinine in cats who survived was 227 μmol/L (range 80 to 304) and was 190 μmol/L (range 150 to 236) in cats who did not survive. Urine protein to creatinine ratio (UPCR) was available for review in five of 15 (33.3%) cases. Median UPCR in dogs and cats was 1.48 (range 0.67 to 18.26) and 0.45 (range 0.41 to 0.5), respectively.

SBP measurements were available for all cases. SBP was initially measured by non‐invasive Doppler in all cases, and one case had an arterial catheter placed for invasive blood pressure (IBP) measurement. Median admission and peak SBP in dogs were 220 (range 190 to 300) and 290 mmHg (range 210 to 300), respectively. Median admission and peak SBP in cats were 230 (range 220 to 300) and 250 mmHg (range 220 to 300), respectively.

### Diagnosis

The most common diagnosis in cats was AKI in six of eight (75%) cases, followed by idiopathic hypertension [one of eight (12.5%)] and hyperthyroidism [one of eight (12.5%)]. The underlying aetiology of AKI in cats was acute‐on‐chronic kidney disease [three of six (50%)], ureteral obstruction [two of six (33.3%)] and renal carcinoma [one of six (16.7%)]. The most common diagnosis in dogs was AKI in three of seven (42.9%) cases, followed by idiopathic hypertension [two of seven (28.6%)], multi‐centric lymphoma [one of seven (14.3%)] and suspected cutaneous and renal glomerular vasculopathy [one of seven (14.3%)]. The underlying aetiology of AKI in dogs was leptospirosis [one of three (33.3%)], suspected intoxication [one of three (33.3%)] and immune‐mediated glomerulonephritis [one of three (33.3%)].

### Clinical management

Medications used to treat the hypertensive crisis are listed in Tables 2 and 3. Three patients received antihypertensive treatment before referral including amlodipine (0.15 to 0.63 mg/kg; n=2) and benazepril (0.5 to 0.6 mg/kg; n=2). Amlodipine was the most commonly used first agent [10 of 15 (66.6%)], followed by hydralazine [four of 15 (26.6%)] and hypertonic saline [one of 15 (6.7%)]. Detailed dose and administration information on hydralazine usage at any point during case management was available for four of six (75%) cases. Hydralazine was given as a bolus (0.16 mg/kg; 0.1 to 0.25) a median of 2.5 (range 1 to 4) times and in two cases was followed by a continuous rate infusion (1 to 5 mcg/kg/minute).

**Table 2:** Medications used in the treatment of canine patients following diagnosis of hypertensive emergency at the Queen Mother Hospital for Animals, Royal Veterinary College between January 1, 2003 and December 31, 2019. Median dose was calculated where administration data including total dose and bodyweight were available

Medication	Number of cases receiving medication (%)	Median initial dose (range)
Amlodipine	6 (85.7)	0.34 mg/kg (0.15 to 1.6; n=4)
Hydralazine	4 (57.1)	0.16 mg/kg (0.12 to 0.2; n=2)
Sodium nitroprusside	3 (42.8)	1 mcg/kg/minute (n=1)
Enalapril	1 (14.3)	0.77 mg/kg (n=1)
Telmisartan	1 (6.7)	0.5 mg/kg (n=1)

**Table 3:** Medications used in the treatment of feline patients following diagnosis of hypertensive emergency at the Queen Mother Hospital for Animals, Royal Veterinary College between January 1, 2003 and December 31, 2019. Median dose was calculated where administration data including total dose and bodyweight were available

Medication	Number of cases (%)	Median initial dose (range)
Amlodipine	8 (100.0)	0.29 mg/kg (0.13 to 1.0; n=8)
Hydralazine	2 (25.0)	0.18 mg/kg (0.1 to 0.25; n=2)
Hypertonic saline	1 (12.5)	5 ml/kg (n=1)
Mannitol	1 (12.5)	0.5 mg/kg (n=1)

A median of two agents was used in all cases. Six cases had full hospital records available for review. Adherence to human guidelines for treatment of hypertensive emergency is summarised in Table 4. Overall in‐hospital mortality was 46.7% (n=7). In‐hospital mortality occurred in three of eight (37.5%) cats of which two of three (66.6%) were euthanased and one of three (33.3%) suffered a cardiopulmonary arrest. In‐hospital mortality occurred in four of seven (57.1%) dogs of which four of four (100%) were euthanased. Of seven cases which did not survive to discharge, five of seven (71.4%) had an AKI of which two of five (40%) were euthanased due to oligoanuria and fluid overload. Of eight cases which survived, median discharge SBP in dogs and cats was 140 (range 125 to 160) and 160 mmHg (range 115 to 170), respectively. Of seven cases which did not survive, median last recorded blood pressure in dogs and cats was 190 (144 to 300) and 200 mmHg (range 170 to 212), respectively.

**Table 4:** Adherence to treatment guidelines for the resolution of hypertensive emergency in patients diagnosed with hypertensive emergency with full information available regarding in‐hospital management between January 1, 2003 and December 31, 2019

Case number	Admit NIBP (mmHg)	NIBP at start of treatment (mmHg)	Next NIBP reading (mmHg)	Time to next NIBP reading (hours)	Time taken to drop NIBP by 25% (hours)	Time taken to drop to or below 160 mmHg (hours)
Dogs						
1	210	210	186	1.5	25	25
2	300	300	180	3	1.9	5.5
3	200	200	220	1	‐	‐
Cats						
4	260	260	160	1	<1	1
5	250	260	180	3	2.4	*
6	300	300	260	1.25	8	13

‐ indicates that Case 3 did not show any response to treatment and had no decrease in NIBP

* indicates that Case 5 did not achieve an NIBP of 160 mmHg or lower before euthanasia

## DISCUSSION

This small retrospective case series is the largest to date reporting the presentation, treatment and outcomes of dogs and cats with hypertensive emergency. Before this study, literature on hypertensive emergency was limited to three small case series on hypertensive encephalopathy including a descriptive postmortem case series of 12 cats with presumed HE (Church *et al*. 2019), a case series of two dogs and two cats without information on case progression (O'Neill *et al*. 2013a,b) and an experimental study following four cats post‐sub‐total nephrectomy (Brown *et al*. 2005). As a result, there are currently no evidence‐based clinical recommendations for treatment goals of hypertensive emergency in veterinary medicine.

Seizures were the most common presenting complaint in our study. Hypertensive encephalopathy results in vasogenic and interstitial oedema that can lead to clinical signs such as seizures and altered mentation due to two main theories: the excessive Baylis effect and “breakthrough theory” (Dinsdale 1982). The Baylis effect refers to vasoconstriction in response to an increase in intraluminal pressure, resulting in decreased cerebral blood flow and ischaemic damage to blood vessels and the blood–brain barrier. Conversely, the breakthrough theory suggests that excessive intraluminal pressure overwhelms the myogenic autoregulatory response and actually results in vasodilation with damage to the blood vessels and blood–brain barrier (Dinsdale 1982). The pathophysiology of hypertensive encephalopathy remains unclear; however, the presence of vasogenic oedema remains a consistent finding in veterinary patients with this condition (Church *et al*. 2019). This study highlights the importance of blood pressure evaluation in patients presenting with seizures and the requirement to manage severe hypertension in a timely manner.

Diagnosing and treating hypertension requires accurate measurement of the patient's blood pressure. Whilst invasive arterial blood pressure measurement via an arterial catheter is considered the gold standard, in veterinary medicine, placement can be technically challenging and is associated with potential risks such as infection, embolus formation and haemorrhage (Bosiack *et al*. 2010). However, most complications do not pose an immediate threat to the patient and do not justify the removal of the catheter (Hagley *et al*. 2021). Only one dog in this study had an arterial catheter placed. This dog was started on hydralazine and had dosage titrated to effect based on continuous IBP measurement. The need for IBP monitoring remains controversial in human medicine with no evidence showing mortality benefit of IBP use over NIBP. Recommendations for arterial line placement for management of hypertensive emergency in people include labile blood pressure and use of sodium nitroprusside (Varon & Marik 2003). Unfortunately, to date, no indirect device has reached the ACVIM validation criteria (Brown *et al*. 2007) and so caution is advised in interpreting results obtained by non‐invasive methods (Acierno *et al*. 2018). Given the low risk associated with placement and maintenance of arterial catheters (Hagley *et al*. 2021) and the potential inaccuracies associated with NIBP in veterinary patients, arterial catheters should be considered more frequently in hypertensive emergency.

A range of therapeutics was used to treat hypertensive emergency in this study. Amlodipine besylate was administered to 14 of 15 (93.3%) patients during hospitalisation. Amlodipine is a calcium channel blocker (CCB) which acts in cardiac and vascular smooth muscle (Cooke & Snyder 1998). Amlodipine is recommended for hypertensive urgency, but not hypertensive emergency, in veterinary medicine (Acierno *et al*. 2018). Similarly, amlodipine is not recommended for use in management of hypertensive emergency in people (Van Den Born *et al*. 2019) although is appropriate for hypertensive urgency and chronic hypertension (Whelton *et al*. 2018), likely owing to its longer time to onset (Abernethy 1992). Instead, clevidipine, a parenteral CCB, is recommended due to its quick onset of action (within 2 minutes), short half‐life (<30 minutes) and superiority over nitroglycerine, sodium nitroprusside and nicardipine in acute hypertension (Aronson *et al*. 2008; Pollack *et al*. 2009; Van Den Born *et al*. 2019). Unfortunately, there are no clinical veterinary studies on its use. Many practices will not have access to these parenteral medications and so oral medications, such as amlodipine, may be the best option available, either before referral or as the primary treatment where referral is not an option. Until larger prospective studies are available, there is currently no evidence to suggest that the use of amlodipine as a first‐line therapy is harmful compared with alternative agents in veterinary medicine. However, assessing an individual patient's response to initial therapy is important to determine the requirement for multi‐modal antihypertensive treatment.

Hydralazine was the second most common agent administered to six of 15 (40%) patients during hospitalisation. Hydralazine causes preferential vasodilation of arteriolar smooth muscle (Jacobs 1984). Hydralazine has been reported for use in postoperative renal transplant cats with hypertension (Kyles *et al*. 1999) and several abstracts are available on its use in congestive heart failure (Kittleson & Hamlin 1983, Kittleson *et al*. 1985). However, there are no clinical veterinary studies on its use in HE, and recommended dosages are extrapolated from human medicine (Acierno *et al*. 2018). In this study, hydralazine was most commonly used as a bolus (0.16 mg/kg; 0.1 to 0.25), with two cases receiving additional drug as a continuous rate infusion (1 to 5mcg/kg/minute). Hydralazine is no longer recommended for use in people with HE due to its unpredictable dose response and prolonged duration of action (Van Den Born *et al*. 2019). Given the lack of appreciable clinical evidence and unknown pharmacokinetics and pharmacodynamics in veterinary patients, caution should be exercised in using hydralazine for control of veterinary HE. However, as for amlodipine, there is no evidence in veterinary medicine to indicate the superiority of hydralazine as a first‐line antihypertensive agent, and pharmacokinetic/pharmacodynamic differences between species must be considered when making treatment recommendations.

Sodium nitroprusside was the third most common agent, administered to three of 15 (20%) patients during hospitalisation. Sodium nitroprusside is a non‐specific vasodilator that has been recommended for use as an adjunctive medication for acute congestive heart failure in dogs with severe mitral regurgitation (Greer *et al*. 2004); however, it has not been evaluated in hypertensive emergency. Although sodium nitroprusside has been shown to be safe in human patients with hypertensive emergency (Hirschl *et al*. 1997) and is recommended as an alternative treatment in the European Society of Cardiology (ESC) Guidelines (Van Den Born *et al*. 2019), administration is not without its risks. Sodium nitroprusside may be difficult to titrate resulting in unwanted “overshoot” hypertension or hypotension (Aronson *et al*. 2008), requires special handling to prevent its degradation from light (Varon & Marik 2003) and prolonged infusion can result in cyanide toxicity (Michenfelder 1977, Pasch *et al*. 1983). Additionally, sodium nitroprusside can lead to a decrease in cerebral blood flow secondary to an increase in intracranial pressure from cerebral vasodilation (Anile *et al*. 1981), making it a less desirable agent for patients with hypertensive encephalopathy.

Hyperosmolar agents were used in one cat that presented with acute onset seizures. Imaging findings in this cat were consistent with diffuse forebrain signs, without evidence supporting raised intracranial pressure, hence its inclusion in this case series. Hyperosmolar agents are recommended for use in the treatment of raised intracranial pressure to reduce the risk of brain herniation (Cook *et al*. 2020). Systemic hypertension can be seen alongside bradycardia in the Cushing reflex (Fodstad *et al*. 2006), complicating decision making in the hypertensive seizuring patient.

Several agents recommended for use in human hypertensive emergency were not used in this study, including labetalol, nicardipine and clevidipine. Labetalol is a combined alpha‐ and beta‐adrenergic blocking parenteral drug that is frequently used in cases of human hypertensive emergency. Labetalol performed favourably in a recent cohort of dogs undergoing surgery with intra‐ and postoperative non‐nociceptive hypertension (Zublena *et al*. 2020). Fenoldopam has been recommended for use in veterinary patients with hypertensive emergency based on its safety profile in patients with AKI (Nielsen *et al*. 2015) and is recommended for use in human hypertensive emergency (Van Den Born *et al*. 2019) although it can be challenging to obtain. Further studies are needed to assess the safety and efficacy of these drugs in veterinary patients with hypertensive emergency. It is possible that lack of use of parenteral medications in this study was due to a lack of familiarity with safety profiles and efficacy.

Treatment goals of hypertensive emergency depend on the type of hypertensive end‐organ damage. For example, patients with acute cardiogenic pulmonary oedema have more rapid targets for a decrease in ABP compared with those with ischaemic stroke (Van Den Born *et al*. 2019). No consensus statement exists in human medicine for treatment of hypertensive emergency, but general guidelines are available as previously mentioned as too aggressive a reduction in ABP may worsen hypoperfusion and target end‐organ damage (Rodriguez *et al*. 2010). Assessment of adherence to these guidelines in our study was limited by the lack of available clinical records. In all but one case, time taken to drop NIBP by 25% was greater than 1 hour suggesting that there is scope for improvement in the timeline of management of hypertensive emergency in veterinary patients.

Unfortunately, only one case was close to adhering to the treatment guidelines with a decrease of 25% in 2 hours, and a subsequent decrease to an SBP of 160 mmHg in 5.5 hours. This cat received hydralazine as a bolus of 0.2 mg/kg followed by 1 to 2mcg/kg/minute over 48 hours before transitioning onto 0.8 mg/kg total daily dose of amlodipine. The cat was discharged successfully with a final NIBP of 120 mmHg and diagnosed with idiopathic hypertension.

Given the lack of consensus in human medicine and the infrequent nature of case presentation, it is not surprising that there was variation in management strategies in our study. It is also currently not known whether these human guidelines should be applied to veterinary patients. An individually tailored approach to each case based on response to antihypertensive medication is important, as some patients may not tolerate rapid changes in blood pressure, and the complexities of the human guidelines serve as an example of how each individual case is unique. Raising awareness of hypertensive emergency and potential treatment goals may help to standardise therapy in future patients, allowing the establishment of veterinary guidelines in future studies.

Overall in‐hospital mortality was 46.7%, most commonly due to euthanasia [six of seven (85.7%)]. The majority of patients who did not survive to discharge had an AKI. AKI has previously been associated with high mortality rates and a recent meta‐analysis found an overall mortality of 53.1% in cats and 45% in dogs, although infectious aetiologies were associated with a lower mortality rate (Legatti *et al*. 2018). Most recently, a retrospective study of 249 dogs found a lower overall mortality rate of 34%, with higher rates of mortality associated with higher grades of AKI (Rimer *et al*. 2022). Similarly, dogs who died in our study tended to have a higher creatinine than those who survived, although the sample size was too small for statistical analysis. Two of the five cases with AKI were euthanased due to oligoanuria and fluid overload, rather than the progression of clinical signs associated with hypertensive emergency. Fluid overload is common during hospitalisation of patients with AKI, demonstrating the necessity for careful management of fluid balance (Cole *et al*. 2020).

## LIMITATIONS

This retrospective case series had several limitations. Most importantly, many cases were excluded due to lack of serial blood pressure measurements in the clinical record resulting in a very small sample size. Unfortunately, it is not possible to determine whether the lack of recording meant a lack of testing, as it is not common for serial blood pressures to be recorded in the final discharge report at the authors' institute. Furthermore, historical hospital kennel sheet records were infrequently available for direct review, limiting the ability to assess adherence to human guidelines for therapy. Due to the retrospective nature of the study, the case recruitment timeline included a period of time when medical records were paper based *versus* more recent transition to computerised medical records. Improved availability of data from more recent cases could therefore contribute to study and population bias. As a result of the small sample size, inferential statistics were not performed due to a lack of statistical power and, as such, conclusions regarding risk factors for mortality could not be evaluated. Due to the small sample size, caution should be exercised in interpreting data regarding drug usage or underlying disease aetiology in this study population. Larger studies would be required to assess a correlation between use of individual agents and impaired patient outcome. Finally, it is possible that the low number of cases was due to poor recognition of hypertensive emergency as a clinical presentation. Further observational studies focusing on prospective enrolment and monitoring of cases with hypertensive emergency are warranted before studies with clinical interventions.

In conclusion, hypertensive emergency is an uncommon presentation in veterinary medicine, but it is associated with high in‐hospital mortality (47.3%) and the majority of patients who did not survive had an AKI. Patients with hypertensive emergency can present with seizures, blindness and/or altered mentation, identifying a need to measure blood pressure in these patients. Oral amlodipine was used commonly as an initial agent despite recommendations for parenteral antihypertensive use in hypertensive emergency. Further studies would be required to establish superiority of parenteral antihypertensive agents as first‐line therapy. In the limited number of patients where full hospital records were available, return to at least a borderline hypertensive category (SBP<160 mmHg) was achieved in 66.6% (four of six) of cases. Veterinary patients may benefit from arterial catheter placement for titration of parenteral antihypertensive medications in line with human guidelines for resolution of hypertensive emergency due to limitations of non‐invasive methods. Future studies should focus on prospective enrolment of cases before establishing treatment protocols.

### Conflict of interest

The authors declared no potential conflicts of interest with respect to the research, authorship and/or publication of this article.

### Author Contributions

**Dave Beeston:** Conceptualization (equal); data curation (lead); formal analysis (lead); investigation (equal); methodology (equal); visualization (equal); writing – original draft (lead); writing – review and editing (equal). **Rosanne Jepson:** Conceptualization (equal); data curation (supporting); formal analysis (supporting); methodology (equal); supervision (lead); visualization (equal); writing – original draft (supporting); writing – review and editing (lead). **Stefano Cortellini:** Conceptualization (equal); data curation (supporting); formal analysis (supporting); methodology (equal); supervision (lead); visualization (equal); writing – original draft (equal); writing – review and editing (lead).

### Ethics statement

This work involved the use of non‐experimental animals only (including owned or unowned animals and data from prospective or retrospective studies). Established internationally recognised high standards (“best practice”) of individual veterinary clinical patient care were followed. Ethical approval from a committee, while not necessarily required, was nonetheless obtained, as stated in the manuscript. Informed consent (either verbal or written) was obtained from the owner or legal. Custodian of all animal(s) described in this work (either experimental or non‐experimental animals) for the procedure(s) undertaken (either prospective or retrospective studies). No animals or humans are identifiable within this publication, and therefore additional informed consent for publication was not required.
